# Bis(4-chloro­benzyl­ammonium) tetra­kis(2,6-diethyl­anilinium) cyclo­hexa­phosphate tetra­hydrate

**DOI:** 10.1107/S1600536809006655

**Published:** 2009-02-28

**Authors:** Olfa Amri, Sonia Abid, Mohamed Rzaigui

**Affiliations:** a Laboratoire de Chimie des Matériaux, Faculté des Sciences de Bizerte, 7021 Zarzouna Bizerte, Tunisia

## Abstract

In the crystal of the title hydrated mol­ecular salt, 2C_7_H_9_ClN^+^·4C_10_H_16_N^+^·P_6_O_18_^6−^·4H_2_O, the packing consists of a three-dimensional O—H⋯O and N—H⋯O hydrogen-bonded network resulting from the association of anionic layers built up from centrosymmetric cyclohexaphosphate ions and water mol­ecules and the two types of organic cations.

## Related literature

For related structures, see: Amri *et al.* (2007 ▶, 2008 ▶); Marouani & Rzaigui (2002 ▶). For background, see: Kresge *et al.* (1992 ▶); Katsoulis (1998 ▶).
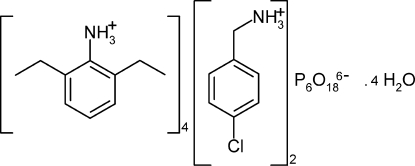


## Experimental

### 

#### Crystal data

2C_7_H_9_ClN^+^·4C_10_H_16_N^+^·P_6_O_18_^6−^·4H_2_O
*M* *_r_* = 1432.04
Monoclinic, 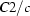

*a* = 31.437 (2) Å
*b* = 14.178 (2) Å
*c* = 16.034 (2) Å
β = 99.60 (2)°
*V* = 7046.5 (14) Å^3^
*Z* = 4
Mo *K*α radiation
μ = 0.30 mm^−1^
*T* = 293 K
0.20 × 0.18 × 0.16 mm

#### Data collection

Enraf–Nonius TurboCAD-4 diffractometer
Absorption correction: none
11637 measured reflections
8452 independent reflections
5321 reflections with *I* > 2σ(*I*)
*R*_int_ = 0.035
2 standard reflections frequency: 120 min intensity decay: 5%

#### Refinement

*R*[*F* ^2^ > 2σ(*F* ^2^)] = 0.053
*wR*(*F* ^2^) = 0.144
*S* = 1.02
8452 reflections
417 parameters
H-atom parameters not refined
Δρ_max_ = 0.57 e Å^−3^
Δρ_min_ = −0.44 e Å^−3^

### 

Data collection: *CAD-4 EXPRESS* (Enraf–Nonius, 1994 ▶); cell refinement: *CAD-4 EXPRESS*; data reduction: *XCAD4* (Harms & Wocadlo, 1995 ▶); program(s) used to solve structure: *SHELXS97* (Sheldrick, 2008 ▶); program(s) used to refine structure: *SHELXL97* (Sheldrick, 2008 ▶); molecular graphics: *ORTEP-3 for Windows* (Farrugia, 1997 ▶); software used to prepare material for publication: *WinGX* (Farrugia, 1999 ▶).

## Supplementary Material

Crystal structure: contains datablocks I, global. DOI: 10.1107/S1600536809006655/hb2918sup1.cif

Structure factors: contains datablocks I. DOI: 10.1107/S1600536809006655/hb2918Isup2.hkl

Additional supplementary materials:  crystallographic information; 3D view; checkCIF report

## Figures and Tables

**Table 1:** Hydrogen-bond geometry (Å, °)

*D*—H⋯*A*	*D*—H	H⋯*A*	*D*⋯*A*	*D*—H⋯*A*
N1—H1*A*⋯O9^i^	0.89	1.80	2.685 (3)	171
N1—H1*B*⋯O2	0.89	2.03	2.758 (3)	138
N1—H1*C*⋯O4	0.89	2.13	2.935 (3)	151
N2—H2*A*⋯O4^ii^	0.89	1.94	2.827 (3)	172
N2—H2*B*⋯O1	0.89	2.05	2.887 (3)	156
N2—H2*C*⋯O7	0.89	1.94	2.809 (3)	166
N3—H3*A*⋯O5^iii^	0.89	1.89	2.762 (3)	165
N3—H3*B*⋯O9	0.89	1.93	2.799 (3)	164
N3—H3*C*⋯O10	0.89	1.99	2.800 (5)	151
O10—H1⋯O11	0.85	2.22	2.923 (6)	140
O10—H2⋯O11^iv^	0.86	2.25	2.831 (5)	125
O11—H6⋯O2^v^	0.85	2.23	2.740 (4)	118
O11—H7⋯O5^iii^	0.85	2.17	2.781 (4)	129

Symmetry codes: (i) 
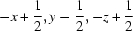
; (ii) 
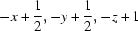
; (iii) 
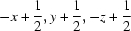
; (iv) 
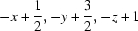
; (v) 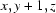.

**Fig. 1.**
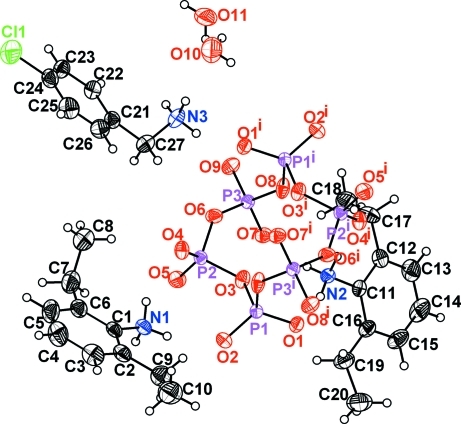
View of (I) with displacement ellipsoids for the non-H atoms drawn at the 30% probability level (symmetry code: (i) 1/2 - x, 1/2 - y, 1 -z).

**Fig. 2.**
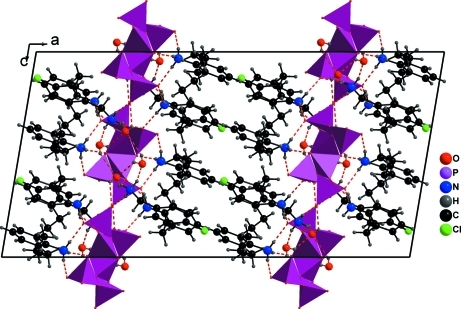
Projection of (I) along b axis.

## supplementary crystallographic information

### Comment

The synthesis and characterization of organic–inorganic solid state hybrid materials has attracted great attention due to their structural diversity (Kresge *et al.*, 1992) and widely promising potential areas in chemistry, biology and material science (Katsoulis, 1998). As part of our studies in this area, we report here synthesis and crystal structure of the the title compound, (I), (Fig. 1).

The centrosymmetric cyclohexaphosphate anion and the two water molecules are linked together by O—H···O hydrogen bonds to form inorganic layers parallel to the (b,c) plane. On both sides of each inorganic layer, are grafted the organic cations compensating their negatives charges (Fig. 2). Inside this arrangement, the geometry of the phosphoric rings is comparable to those found in [2,6-(CH_3_)_2_C_6_H_3_NH_3_]_4_[2,6-(C_2_H_5_)_2_C_6_H_3_NH_3_]\ _2_P_6_O_18_.4H_2_O (Amri *et al.* 2007) and (1,6NH_3_C_6_H_12_NH_3_)(C_6_H_5_NH_3_)_4_P_6_O_18_.6H_2_O (Marouani *et al.* 2002). In the title compound, the phosphoric rings have the same internal symmetry and thus built up by only three independent PO_4_tetrahedra P1O_4_, P2O_4_ and P3O_4_. In the PO_4 _tetrahedra, the P—O distances range in [1.470 (3) - 1.610 (2) Å] and the O—P—O bond angles in [99.4 (2) - 121.2 (2)°]. It is the same for the P—P distances ranging from 2.901 (1) and 2.937 (1) Å which are comparable to values generally measured. For the organic cations, the main features measured are similar to distances and angles usually reported for such molecules (Amri *et al.*2007, Amri *et al.* 2008). The phenyl rings of these groups are almost planar, with mean deviations of ± 0.004 and ± 0.011Å for 2,6- diethylphenylammonium and ± 0.003 Å for 4-chlorobenzylanilinium.

### Experimental

Cyclohexaphosphoric acid (4.8 mmol) was slowly added to an ethanolic solution of 2,6-diethylaniline (3.16 ml; 19.2 mmol) and 4-chlorobenzylaniline (1.16 ml, 9.6 mmol) in a molar ratio of 2:1 respectively. The obtained solution was slowly evaporated at room temperature. After some days, colourless prisms of (I) were formed. The cyclohexaphosphoric acid is freshly prepared by passing a solution of Li_6_P_6_O_18_(3 g, 4.8 mmol) through an ion exchange resin in its H-state (Amberlite IR 120).

### Refinement

The water H atoms were located in a difference map and freely refined. The other H atoms were positioned geometrically (N—H = 0.89, C—H = 0.93–0.96 Å) and refined as riding with *U*_iso_(H) = 1.2U_eq_(C,N) or 1.5U_eq_(methyl C).

### Crystal data

**Table:**

2C_7_H_9_ClN^+^·4C_10_H_16_N^+^·P_6_O_18_^6^^−^·4H_2_O	*F*(000) = 3024
*M**_r_* = 1432.04	*D*_x_ = 1.350 Mg m^−^^3^
Monoclinic, *C*2/*c*	Mo *K*α radiation, λ = 0.71073 Å
Hall symbol: -C 2yc	Cell parameters from 25 reflections
*a* = 31.437 (2) Å	θ = 9–11°
*b* = 14.178 (2) Å	µ = 0.30 mm^−^^1^
*c* = 16.034 (2) Å	*T* = 293 K
β = 99.60 (2)°	Prism, colourless
*V* = 7046.5 (14) Å^3^	0.20 × 0.18 × 0.16 mm
*Z* = 4	

### Data collection

**Table:**

Enraf–Nonius TurboCAD-4 diffractometer	*R*_int_ = 0.035
Radiation source: Enraf Nonius FR590	θ_max_ = 28.0°, θ_min_ = 2.1°
graphite	*h* = −41→41
non–profiled ω scans	*k* = 0→18
11637 measured reflections	*l* = −5→21
8452 independent reflections	2 standard reflections every 120 min
5321 reflections with *I* > 2σ(*I*)	intensity decay: 5%

### Refinement

**Table:**

Refinement on *F*^2^	Primary atom site location: structure-invariant direct methods
Least-squares matrix: full	Secondary atom site location: difference Fourier map
*R*[*F*^2^ > 2σ(*F*^2^)] = 0.053	Hydrogen site location: inferred from neighbouring sites
*wR*(*F*^2^) = 0.144	H-atom parameters not refined
*S* = 1.02	*w* = 1/[σ^2^(*F*_o_^2^) + (0.0697*P*)^2^ + 2.2936*P*] where *P* = (*F*_o_^2^ + 2*F*_c_^2^)/3
8452 reflections	(Δ/σ)_max_ < 0.001
417 parameters	Δρ_max_ = 0.56 e Å^−^^3^
0 restraints	Δρ_min_ = −0.44 e Å^−^^3^

### Special details

**Table:**

Geometry. All e.s.d.'s (except the e.s.d. in the dihedral angle between two l.s. planes) are estimated using the full covariance matrix. The cell e.s.d.'s are taken into account individually in the estimation of e.s.d.'s in distances, angles and torsion angles; correlations between e.s.d.'s in cell parameters are only used when they are defined by crystal symmetry. An approximate (isotropic) treatment of cell e.s.d.'s is used for estimating e.s.d.'s involving l.s. planes.
Refinement. Refinement of *F*^2^ against ALL reflections. The weighted *R*-factor *wR* and goodness of fit *S* are based on *F*^2^, conventional *R*-factors *R* are based on *F*, with *F* set to zero for negative *F*^2^. The threshold expression of *F*^2^ > σ(*F*^2^) is used only for calculating *R*-factors(gt) *etc*. and is not relevant to the choice of reflections for refinement. *R*-factors based on *F*^2^ are statistically about twice as large as those based on *F*, and *R*- factors based on ALL data will be even larger.

### Fractional atomic coordinates and isotropic or equivalent isotropic displacement parameters (Å^2^)

**Table:**

	*x*	*y*	*z*	*U*_iso_*/*U*_eq_	
Cl1	0.01382 (3)	0.69163 (8)	0.12825 (8)	0.0880 (3)	
P1	0.24807 (2)	0.07584 (5)	0.44736 (4)	0.03164 (16)	
P2	0.23420 (2)	0.23566 (5)	0.33302 (4)	0.03092 (15)	
P3	0.293786 (19)	0.38486 (4)	0.40635 (4)	0.03042 (15)	
O1	0.28646 (5)	0.04571 (12)	0.50699 (11)	0.0400 (4)	
O2	0.22756 (7)	0.01330 (14)	0.37887 (13)	0.0541 (5)	
O3	0.26078 (5)	0.17399 (13)	0.40739 (12)	0.0414 (4)	
O4	0.18744 (5)	0.22936 (14)	0.33497 (12)	0.0428 (4)	
O5	0.24898 (7)	0.21336 (15)	0.25302 (12)	0.0524 (5)	
O6	0.24927 (6)	0.33887 (13)	0.36243 (14)	0.0485 (5)	
O7	0.33084 (5)	0.32302 (13)	0.39994 (12)	0.0423 (4)	
O8	0.28898 (6)	0.38724 (15)	0.50341 (11)	0.0462 (5)	
O9	0.29240 (6)	0.48264 (13)	0.37325 (13)	0.0454 (5)	
O10	0.19365 (13)	0.6949 (3)	0.4542 (2)	0.1059 (10)	
H1	0.2147	0.7334	0.4620	0.21 (4)*	
H2	0.1988	0.6545	0.4948	0.15 (3)*	
O11	0.25125 (15)	0.8271 (2)	0.38834 (19)	0.1238 (15)	
H6	0.2411	0.8642	0.3478	0.37 (7)*	
H7	0.2659	0.7851	0.3679	0.58 (12)*	
N1	0.16392 (7)	0.05296 (16)	0.24302 (14)	0.0422 (5)	
H1A	0.1792	0.0362	0.2034	0.063*	
H1B	0.1734	0.0214	0.2905	0.063*	
H1C	0.1669	0.1146	0.2527	0.063*	
N2	0.35074 (6)	0.19299 (15)	0.53288 (14)	0.0363 (5)	
H2A	0.3395	0.2228	0.5729	0.054*	
H2B	0.3377	0.1376	0.5221	0.054*	
H2C	0.3470	0.2278	0.4860	0.054*	
N3	0.21272 (7)	0.57295 (18)	0.32809 (16)	0.0500 (6)	
H3A	0.2205	0.6199	0.2970	0.075*	
H3B	0.2360	0.5407	0.3512	0.075*	
H3C	0.1998	0.5965	0.3688	0.075*	
C1	0.11824 (8)	0.0310 (2)	0.21416 (17)	0.0415 (6)	
C2	0.10027 (9)	−0.0469 (2)	0.24718 (19)	0.0507 (7)	
C3	0.05691 (11)	−0.0647 (3)	0.2167 (2)	0.0688 (10)	
H3	0.0436	−0.1163	0.2373	0.083*	
C4	0.03350 (11)	−0.0076 (3)	0.1571 (3)	0.0799 (12)	
H4	0.0045	−0.0206	0.1382	0.096*	
C5	0.05230 (11)	0.0683 (3)	0.1251 (2)	0.0753 (11)	
H5	0.0360	0.1059	0.0843	0.090*	
C6	0.09543 (10)	0.0899 (2)	0.15295 (19)	0.0530 (8)	
C7	0.11533 (14)	0.1759 (3)	0.1192 (3)	0.0777 (11)	
H7A	0.1459	0.1645	0.1207	0.093*	
H7B	0.1023	0.1853	0.0606	0.093*	
C8	0.10995 (18)	0.2644 (3)	0.1678 (3)	0.1045 (16)	
H8A	0.0799	0.2806	0.1609	0.157*	
H8B	0.1256	0.3149	0.1470	0.157*	
H8C	0.1209	0.2543	0.2267	0.157*	
C9	0.12659 (12)	−0.1088 (3)	0.3131 (2)	0.0683 (10)	
H9A	0.1434	−0.0686	0.3551	0.082*	
H9B	0.1467	−0.1448	0.2860	0.082*	
C10	0.10082 (16)	−0.1772 (3)	0.3583 (3)	0.0936 (13)	
H10A	0.0811	−0.1425	0.3864	0.140*	
H10B	0.1202	−0.2128	0.3991	0.140*	
H10C	0.0849	−0.2194	0.3177	0.140*	
C11	0.39714 (8)	0.17773 (19)	0.56208 (17)	0.0378 (6)	
C12	0.42153 (9)	0.2542 (2)	0.59790 (19)	0.0459 (7)	
C13	0.46474 (11)	0.2379 (3)	0.6290 (2)	0.0671 (10)	
H13	0.4818	0.2869	0.6545	0.081*	
C14	0.48285 (10)	0.1505 (3)	0.6226 (3)	0.0786 (12)	
H14	0.5119	0.1412	0.6438	0.094*	
C15	0.45849 (10)	0.0770 (3)	0.5855 (2)	0.0669 (10)	
H15	0.4713	0.0186	0.5808	0.080*	
C16	0.41455 (9)	0.0887 (2)	0.5545 (2)	0.0461 (7)	
C17	0.40416 (10)	0.3535 (2)	0.6000 (2)	0.0547 (8)	
H17A	0.3729	0.3512	0.5935	0.066*	
H17B	0.4150	0.3817	0.6545	0.066*	
C18	0.41691 (12)	0.4142 (2)	0.5311 (2)	0.0675 (10)	
H18A	0.4478	0.4147	0.5360	0.101*	
H18B	0.4067	0.4773	0.5364	0.101*	
H18C	0.4044	0.3891	0.4769	0.101*	
C19	0.38797 (10)	0.0071 (2)	0.5151 (3)	0.0635 (9)	
H19A	0.3622	0.0033	0.5407	0.076*	
H19B	0.3788	0.0210	0.4555	0.076*	
C20	0.40905 (15)	−0.0872 (3)	0.5220 (3)	0.0992 (15)	
H20A	0.4322	−0.0877	0.4897	0.149*	
H20B	0.3882	−0.1346	0.5006	0.149*	
H20C	0.4203	−0.1002	0.5803	0.149*	
C21	0.14034 (9)	0.5570 (2)	0.23882 (18)	0.0453 (7)	
C22	0.13925 (9)	0.6432 (2)	0.19784 (18)	0.0470 (7)	
H22	0.1651	0.6734	0.1935	0.056*	
C23	0.10094 (9)	0.6850 (2)	0.16347 (19)	0.0488 (7)	
H23	0.1007	0.7427	0.1358	0.059*	
C24	0.06256 (9)	0.6395 (2)	0.1708 (2)	0.0535 (8)	
C25	0.06304 (11)	0.5540 (3)	0.2110 (2)	0.0649 (9)	
H25	0.0372	0.5238	0.2154	0.078*	
C26	0.10184 (11)	0.5128 (2)	0.2447 (2)	0.0579 (8)	
H26	0.1021	0.4548	0.2718	0.070*	
C27	0.18274 (10)	0.5098 (2)	0.2740 (2)	0.0574 (8)	
H27A	0.1771	0.4550	0.3067	0.069*	
H27B	0.1963	0.4881	0.2274	0.069*	

### Atomic displacement parameters (Å^2^)

**Table:**

	*U*^11^	*U*^22^	*U*^33^	*U*^12^	*U*^13^	*U*^23^
Cl1	0.0443 (4)	0.0973 (8)	0.1139 (9)	0.0033 (5)	−0.0115 (5)	−0.0025 (7)
P1	0.0321 (3)	0.0326 (3)	0.0294 (3)	−0.0001 (3)	0.0026 (3)	0.0001 (3)
P2	0.0332 (3)	0.0313 (3)	0.0282 (3)	−0.0024 (3)	0.0053 (2)	0.0001 (3)
P3	0.0303 (3)	0.0308 (3)	0.0303 (3)	−0.0005 (2)	0.0055 (2)	0.0023 (3)
O1	0.0369 (9)	0.0413 (10)	0.0400 (10)	0.0045 (8)	0.0013 (8)	0.0042 (8)
O2	0.0625 (13)	0.0426 (11)	0.0508 (12)	0.0012 (10)	−0.0090 (10)	−0.0127 (10)
O3	0.0355 (9)	0.0458 (10)	0.0419 (10)	−0.0050 (8)	0.0034 (8)	0.0130 (9)
O4	0.0337 (9)	0.0540 (11)	0.0390 (10)	−0.0053 (8)	0.0009 (8)	0.0041 (9)
O5	0.0746 (13)	0.0520 (12)	0.0342 (10)	0.0114 (10)	0.0196 (10)	−0.0005 (9)
O6	0.0363 (9)	0.0353 (10)	0.0686 (14)	0.0020 (8)	−0.0063 (9)	−0.0110 (10)
O7	0.0352 (9)	0.0416 (10)	0.0509 (11)	0.0047 (8)	0.0091 (8)	−0.0015 (9)
O8	0.0359 (9)	0.0754 (14)	0.0281 (9)	0.0093 (9)	0.0074 (8)	0.0112 (9)
O9	0.0457 (10)	0.0401 (10)	0.0529 (12)	0.0012 (8)	0.0149 (9)	0.0137 (9)
O10	0.110 (3)	0.124 (3)	0.077 (2)	0.029 (2)	−0.0021 (17)	−0.005 (2)
O11	0.245 (5)	0.0640 (17)	0.0534 (16)	0.041 (2)	−0.001 (2)	−0.0106 (15)
N1	0.0381 (11)	0.0459 (13)	0.0421 (13)	0.0004 (10)	0.0054 (10)	−0.0121 (11)
N2	0.0335 (10)	0.0355 (11)	0.0391 (12)	0.0028 (9)	0.0036 (9)	−0.0012 (10)
N3	0.0403 (12)	0.0528 (15)	0.0560 (15)	0.0045 (11)	0.0052 (11)	0.0182 (13)
C1	0.0349 (13)	0.0500 (16)	0.0398 (15)	0.0021 (12)	0.0070 (11)	−0.0160 (13)
C2	0.0473 (16)	0.0577 (18)	0.0482 (17)	−0.0016 (14)	0.0107 (14)	−0.0150 (15)
C3	0.0480 (18)	0.079 (2)	0.082 (3)	−0.0122 (18)	0.0180 (18)	−0.010 (2)
C4	0.0417 (18)	0.110 (3)	0.084 (3)	−0.006 (2)	−0.0015 (19)	−0.011 (3)
C5	0.0507 (19)	0.104 (3)	0.066 (2)	0.018 (2)	−0.0057 (17)	−0.006 (2)
C6	0.0488 (16)	0.064 (2)	0.0454 (17)	0.0071 (15)	0.0059 (14)	−0.0089 (16)
C7	0.088 (3)	0.077 (3)	0.063 (2)	0.001 (2)	−0.002 (2)	0.014 (2)
C8	0.128 (4)	0.077 (3)	0.096 (3)	0.013 (3)	−0.019 (3)	0.003 (3)
C9	0.068 (2)	0.062 (2)	0.074 (2)	−0.0057 (18)	0.0119 (19)	0.0038 (19)
C10	0.121 (4)	0.079 (3)	0.084 (3)	−0.007 (3)	0.025 (3)	0.007 (2)
C11	0.0330 (12)	0.0441 (15)	0.0357 (14)	0.0007 (11)	0.0037 (11)	0.0040 (12)
C12	0.0408 (14)	0.0540 (18)	0.0434 (16)	−0.0078 (13)	0.0081 (12)	−0.0010 (14)
C13	0.0469 (18)	0.073 (2)	0.076 (2)	−0.0179 (17)	−0.0053 (16)	0.001 (2)
C14	0.0331 (16)	0.083 (3)	0.112 (3)	−0.0008 (17)	−0.0095 (18)	0.017 (2)
C15	0.0386 (16)	0.061 (2)	0.099 (3)	0.0131 (15)	0.0040 (17)	0.012 (2)
C16	0.0367 (13)	0.0446 (16)	0.0562 (18)	0.0054 (12)	0.0053 (13)	0.0064 (14)
C17	0.0558 (18)	0.0472 (17)	0.063 (2)	−0.0112 (14)	0.0162 (16)	−0.0176 (16)
C18	0.069 (2)	0.055 (2)	0.078 (2)	−0.0131 (17)	0.0102 (19)	−0.0027 (19)
C19	0.0516 (18)	0.0449 (18)	0.091 (3)	0.0065 (14)	0.0014 (18)	−0.0006 (18)
C20	0.096 (3)	0.062 (2)	0.134 (4)	0.014 (2)	0.000 (3)	−0.017 (3)
C21	0.0502 (16)	0.0464 (16)	0.0386 (15)	−0.0045 (13)	0.0054 (13)	0.0021 (13)
C22	0.0412 (14)	0.0513 (17)	0.0488 (17)	−0.0047 (13)	0.0086 (13)	0.0067 (14)
C23	0.0460 (15)	0.0502 (17)	0.0491 (17)	−0.0025 (13)	0.0048 (13)	0.0070 (14)
C24	0.0416 (15)	0.062 (2)	0.0537 (19)	−0.0031 (14)	−0.0010 (14)	−0.0070 (16)
C25	0.0508 (18)	0.068 (2)	0.075 (2)	−0.0224 (17)	0.0086 (17)	−0.0006 (19)
C26	0.064 (2)	0.0505 (18)	0.059 (2)	−0.0121 (16)	0.0089 (16)	0.0057 (16)
C27	0.0620 (19)	0.0477 (18)	0.061 (2)	0.0079 (15)	0.0047 (16)	0.0074 (16)

### Geometric parameters (Å, °)

**Table:**

O7—P3	1.4751 (18)	C1—C6	1.393 (4)
O6—P2	1.5854 (19)	C2—C3	1.392 (4)
O6—P3	1.5970 (19)	C2—C9	1.510 (5)
O1—P1	1.4724 (18)	C6—C5	1.389 (5)
O3—P2	1.5975 (19)	C6—C7	1.512 (5)
O3—P1	1.6101 (19)	C5—C4	1.368 (6)
O4—P2	1.4783 (18)	C5—H5	0.9300
O8—P3	1.5889 (18)	C3—C4	1.369 (5)
O8—P1^i^	1.6003 (19)	C3—H3	0.9300
O9—P3	1.4825 (19)	C4—H4	0.9300
O5—P2	1.470 (2)	C9—C10	1.522 (5)
O2—P1	1.474 (2)	C9—H9A	0.9700
P1—O8^i^	1.6003 (19)	C9—H9B	0.9700
N2—C11	1.472 (3)	C7—C8	1.501 (6)
N2—H2A	0.8900	C7—H7A	0.9700
N2—H2B	0.8900	C7—H7B	0.9700
N2—H2C	0.8900	C10—H10A	0.9600
C11—C16	1.389 (4)	C10—H10B	0.9600
C11—C12	1.395 (4)	C10—H10C	0.9600
C12—C13	1.386 (4)	C8—H8A	0.9600
C12—C17	1.512 (4)	C8—H8B	0.9600
C16—C15	1.398 (4)	C8—H8C	0.9600
C16—C19	1.503 (4)	Cl1—C24	1.735 (3)
C17—C18	1.506 (5)	N3—C27	1.473 (4)
C17—H17A	0.9700	N3—H3A	0.8900
C17—H17B	0.9700	N3—H3B	0.8900
C19—C20	1.489 (5)	N3—H3C	0.8900
C19—H19A	0.9700	C23—C22	1.372 (4)
C19—H19B	0.9700	C23—C24	1.390 (4)
C15—C14	1.369 (5)	C23—H23	0.9300
C15—H15	0.9300	C22—C21	1.385 (4)
C14—C13	1.374 (5)	C22—H22	0.9300
C14—H14	0.9300	C21—C26	1.380 (4)
C13—H13	0.9300	C21—C27	1.514 (4)
C18—H18A	0.9600	C24—C25	1.372 (5)
C18—H18B	0.9600	C25—C26	1.379 (5)
C18—H18C	0.9600	C25—H25	0.9300
C20—H20A	0.9600	C27—H27A	0.9700
C20—H20B	0.9600	C27—H27B	0.9700
C20—H20C	0.9600	C26—H26	0.9300
N1—C1	1.467 (3)	O11—H6	0.8600
N1—H1A	0.8900	O11—H7	0.8500
N1—H1B	0.8900	O10—H1	0.8500
N1—H1C	0.8900	O10—H2	0.8600
C1—C2	1.385 (4)		
			
P2—O6—P3	134.79 (12)	C2—C1—C6	123.5 (3)
P2—O3—P1	129.53 (11)	C2—C1—N1	119.3 (3)
P3—O8—P1^i^	133.32 (12)	C6—C1—N1	117.2 (3)
O5—P2—O4	117.76 (12)	C1—C2—C3	116.7 (3)
O5—P2—O6	109.61 (12)	C1—C2—C9	121.3 (3)
O4—P2—O6	107.36 (11)	C3—C2—C9	122.0 (3)
O5—P2—O3	109.36 (11)	C5—C6—C1	117.0 (3)
O4—P2—O3	110.45 (10)	C5—C6—C7	120.2 (3)
O6—P2—O3	100.95 (10)	C1—C6—C7	122.7 (3)
O7—P3—O9	120.48 (11)	C4—C5—C6	120.8 (4)
O7—P3—O8	106.62 (11)	C4—C5—H5	119.6
O9—P3—O8	109.20 (12)	C6—C5—H5	119.6
O7—P3—O6	111.75 (11)	C4—C3—C2	121.2 (4)
O9—P3—O6	104.64 (11)	C4—C3—H3	119.4
O8—P3—O6	102.77 (11)	C2—C3—H3	119.4
O1—P1—O2	121.24 (11)	C5—C4—C3	120.7 (3)
O1—P1—O8^i^	111.08 (10)	C5—C4—H4	119.6
O2—P1—O8^i^	107.48 (12)	C3—C4—H4	119.6
O1—P1—O3	106.16 (10)	C2—C9—C10	115.5 (3)
O2—P1—O3	109.28 (11)	C2—C9—H9A	108.4
O8^i^—P1—O3	99.39 (11)	C10—C9—H9A	108.4
C11—N2—H2A	109.5	C2—C9—H9B	108.4
C11—N2—H2B	109.5	C10—C9—H9B	108.4
H2A—N2—H2B	109.5	H9A—C9—H9B	107.5
C11—N2—H2C	109.5	C8—C7—C6	113.5 (4)
H2A—N2—H2C	109.5	C8—C7—H7A	108.9
H2B—N2—H2C	109.5	C6—C7—H7A	108.9
C16—C11—C12	123.0 (2)	C8—C7—H7B	108.9
C16—C11—N2	119.3 (2)	C6—C7—H7B	108.9
C12—C11—N2	117.7 (2)	H7A—C7—H7B	107.7
C13—C12—C11	117.2 (3)	C9—C10—H10A	109.5
C13—C12—C17	118.9 (3)	C9—C10—H10B	109.5
C11—C12—C17	123.8 (3)	H10A—C10—H10B	109.5
C11—C16—C15	117.2 (3)	C9—C10—H10C	109.5
C11—C16—C19	122.4 (2)	H10A—C10—H10C	109.5
C15—C16—C19	120.3 (3)	H10B—C10—H10C	109.5
C18—C17—C12	112.0 (3)	C7—C8—H8A	109.5
C18—C17—H17A	109.2	C7—C8—H8B	109.5
C12—C17—H17A	109.2	H8A—C8—H8B	109.5
C18—C17—H17B	109.2	C7—C8—H8C	109.5
C12—C17—H17B	109.2	H8A—C8—H8C	109.5
H17A—C17—H17B	107.9	H8B—C8—H8C	109.5
C20—C19—C16	116.8 (3)	C27—N3—H3A	109.5
C20—C19—H19A	108.1	C27—N3—H3B	109.5
C16—C19—H19A	108.1	H3A—N3—H3B	109.5
C20—C19—H19B	108.1	C27—N3—H3C	109.5
C16—C19—H19B	108.1	H3A—N3—H3C	109.5
H19A—C19—H19B	107.3	H3B—N3—H3C	109.5
C14—C15—C16	120.8 (3)	C22—C23—C24	118.8 (3)
C14—C15—H15	119.6	C22—C23—H23	120.6
C16—C15—H15	119.6	C24—C23—H23	120.6
C15—C14—C13	120.7 (3)	C23—C22—C21	121.4 (3)
C15—C14—H14	119.7	C23—C22—H22	119.3
C13—C14—H14	119.7	C21—C22—H22	119.3
C14—C13—C12	121.1 (3)	C26—C21—C22	118.7 (3)
C14—C13—H13	119.4	C26—C21—C27	120.1 (3)
C12—C13—H13	119.4	C22—C21—C27	121.2 (3)
C17—C18—H18A	109.5	C25—C24—C23	120.5 (3)
C17—C18—H18B	109.5	C25—C24—Cl1	120.1 (3)
H18A—C18—H18B	109.5	C23—C24—Cl1	119.4 (3)
C17—C18—H18C	109.5	C24—C25—C26	119.9 (3)
H18A—C18—H18C	109.5	C24—C25—H25	120.1
H18B—C18—H18C	109.5	C26—C25—H25	120.1
C19—C20—H20A	109.5	N3—C27—C21	112.8 (2)
C19—C20—H20B	109.5	N3—C27—H27A	109.0
H20A—C20—H20B	109.5	C21—C27—H27A	109.0
C19—C20—H20C	109.5	N3—C27—H27B	109.0
H20A—C20—H20C	109.5	C21—C27—H27B	109.0
H20B—C20—H20C	109.5	H27A—C27—H27B	107.8
C1—N1—H1A	109.5	C25—C26—C21	120.6 (3)
C1—N1—H1B	109.5	C25—C26—H26	119.7
H1A—N1—H1B	109.5	C21—C26—H26	119.7
C1—N1—H1C	109.5	H6—O11—H7	107.00
H1A—N1—H1C	109.5	H1—O10—H2	106.00
H1B—N1—H1C	109.5		

Symmetry codes: (i) −*x*+1/2, −*y*+1/2, −*z*+1.

### Hydrogen-bond geometry (Å, °)

**Table:**

*D*—H···*A*	*D*—H	H···*A*	*D*···*A*	*D*—H···*A*
N1—H1A···O9^ii^	0.89	1.80	2.685 (3)	171
N1—H1B···O2	0.89	2.03	2.758 (3)	138
N1—H1C···O4	0.89	2.13	2.935 (3)	151
N2—H2A···O4^i^	0.89	1.94	2.827 (3)	172
N2—H2B···O1	0.89	2.05	2.887 (3)	156
N2—H2C···O7	0.89	1.94	2.809 (3)	166
N3—H3A···O5^iii^	0.89	1.89	2.762 (3)	165
N3—H3B···O9	0.89	1.93	2.799 (3)	164
N3—H3C···O10	0.89	1.99	2.800 (5)	151
O10—H1···O11	0.85	2.22	2.923 (6)	140
O10—H2···O11^iv^	0.86	2.25	2.831 (5)	125
O11—H6···O2^v^	0.85	2.23	2.740 (4)	118
O11—H7···O5^iii^	0.85	2.17	2.781 (4)	129

Symmetry codes: (ii) −*x*+1/2, *y*−1/2, −*z*+1/2; (i) −*x*+1/2, −*y*+1/2, −*z*+1; (iii) −*x*+1/2, *y*+1/2, −*z*+1/2; (iv) −*x*+1/2, −*y*+3/2, −*z*+1; (v) *x*, *y*+1, *z*.
